# Idiopathic Inflammatory Myopathy—Molecular Mechanisms Underlying Its Pathogenesis and Physical Therapy Effects

**DOI:** 10.3390/ijms26178369

**Published:** 2025-08-28

**Authors:** Aleksandra Markowska, Beata Tarnacka

**Affiliations:** 1 Department of Rehabilitation Medicine, Faculty of Medicine, Medical University of Warsaw, Spartanska 1, 02-637 Warsaw, Poland; beata.tarnacka@wum.edu.pl; 2 Doctoral School, The Medical University of Warsaw, 61 Zwirki I Wigury Street, 02-091 Warsaw, Poland

**Keywords:** idiopathic inflammatory myopathy, rare disease, physical therapy, mitochondrial dysfunctions, myokines, endoplasmic reticulum stress, necroptosis, neutrophil extracellular traps, damage-associated molecular pattern molecules, precision medicine

## Abstract

Idiopathic inflammatory myopathies (IIMs) are rare autoimmune disorders characterized by muscle weakness. As currently used immunosuppressive treatment presents several limitations, recent investigations focus on elucidating immune and nonimmune molecular mechanisms underlying its pathogenesis. Mitochondrial dysfunctions, endoplasmic reticulum stress, neutrophil dysregulation, and alterations in myokines and cell death pathways have been implicated in IIM pathophysiology. In this paper, the newest therapeutic strategies targeting reactive oxygen species overproduction, neutrophil extracellular traps formation, and pyroptotic and necroptotic pathways together with mitochondrial transplantations will be presented, and their safety and efficacy will be discussed. As physical therapy constitutes an essential part of IIM management, an additional focus will be directed towards molecular mechanisms underlying the effects of exercises in myositis treatment. Furthermore, the interplay between immune and nonimmune mechanisms will be analyzed and the translational challenges and limitations of current studies will be investigated.

## 1. Introduction

### 1.1. Idiopathic Inflammatory Myopathies Characteristics

Idiopathic inflammatory myopathies (IIMs) are rare autoimmune disorders characterized primarily by muscle weakness. However, other organs such as lungs, esophagus, and heart may also be affected [1], indicating that IIM should be regarded as systemic inflammatory disorders. Typically, muscle involvement is proximal and symmetrical with the predilection for hip and shoulder girdle muscles and neck flexors. Patients often experience difficulties in climbing stairs, standing up from a seated position, washing their hair or reaching for objects on high shelves. Myalgia may also accompany the clinical presentation. Weaknesses of pharyngeal, esophageal and laryngeal muscles may occur, potentially leading to dysphagia and dysphonia [2]. Idiopathic inflammatory myopathies have been historically classified according to the Bohan and Peter criteria into polymyositis (PM) and dermatomyositis (DM), based on their clinical, laboratory, electrophysiological and pathological features, with typical cutaneous changes differentiating DM from PM [3,4,5]. Skin lesions, characteristic for DM, include reddish-purple rash on eyelids (heliotrope rash), violet papules most commonly found on metacarpophalangeal and interphalangeal joints (Gottron’s papules), erythematous macules on the extensor surfaces (Gottron’s sign), erythematous rash over the neck and shoulder (shawl sign) and lateral parts of hips and thighs (holster sign), periungual redness and telangiectasias as well as hyperkeratosis of fingers and palms (mechanic’s hands) [6,7] [Table 1]. However, since their publication in 1975, other IIM subtypes have been identified. In 1995, the Griggs criteria were established for the diagnosis of inclusion body myositis (IBM). Contrary to other IIM subtypes, IBM is characterized with an asymmetric pattern of weakness, often affecting distal muscles such as finger flexors, wrist flexors, ankle dorsiflexors, as well as knee extensors [6,8]. Histopathological features include mitochondrial dysfunction; inflammatory CD 8+ T cells infiltrate and abnormal aggregation of proteins such as beta amyloid (Aβ), tau protein, or α-synuclein proteins present in neurodegenerative disorders such as Alzheimer’s disease or Parkinson’s disease [9,10] [Table 1]. The course of the disease has a slow progression with weakness developing over the years, in contrast to the acute or subacute onset observed in other IIM subtypes [10]. The disease is more common among the men and usually manifests after the age of 50 whereas other IIM types affect predominantly women [6]. Importantly, immunosuppression treatment does not benefit IBM patients [10].

Recent advances in identification of myositis-specific autoantibodies (MSAs) and myositis-associated autoantibodies (MAAs) have helped diagnosis, classification, and treatment prediction of different IIM subtypes [11,12,13]. More than 60% of patients with myositis have detectable MSA in their serum [14]. In the case of DM, this proportion is estimated to range between 80 and 90% [14]. Anti-Mi-2 (anti-nuclear helicase), anti-MDA-5 (anti-melanoma differentiation-associated gene 5), anti-NXP-2 (anti-nuclear matrix protein 2), anti-TIF-1γ (anti-transcriptional intermediary factor 1), and anti-SAE-1/2 (anti-small ubiquitin-like modifier-1 activating enzyme) are MSA most commonly associated with DM. Among them, anti-Mi-2 is related to typical cutaneous changes, high creatine kinase (CK) levels, and good response to immunosuppressant treatment, while MDA-5 is associated with cutaneous ulcers, alopecia, interstitial lung disease (ILD), and clinically amyopathic disease [11,14,15]. Different subtypes of IIM vary in immunosuppression responsiveness, for example, MDA-5 positive DM requires early and aggressive immunosuppressive combination treatment such as the combination of tacrolimus, rituximab, and glucocorticoids [16] or calcineurin inhibitors, cyclophosphamide, and glucocorticoids [17].

Identification of anti-signal recognition particle (anti-SRP) and, subsequently, anti-3-hydroxy-3-methylglutaryl-coenzyme A reductase (anti-HMGCR) as MSA enabled the recognition of immune-mediated necrotizing myopathy (IMNM) [18], a distinct IIM subtype characterized by prominent muscle atrophy, severe proximal weakness, significantly elevated CK, and the presence of necrotic fibers in histopathological examination [2,18,19] [Table 1]. Patients with anti-SRP antibodies exhibit more severe phenotype with more pronounced muscle weakness and neurological symptoms than those with anti-HMGCR [20], and even among patients who regained muscle strength, most required ongoing immunosuppressive treatment, and their CK levels remained persistently elevated [21].

Discovery of anti-aminoacyl transfer RNA synthetase (ARS) autoantibodies such as anti-Jo-1 (anti-histidyl tRNA synthetase) allowed the classification of new IIM subset, antisynthetase syndrome (ASS) [11] [Table 1]. ASS is classified by some authors into overlap myositis (OM) [6,8], muscle inflammation associated with connective tissue diseases such as Sjögren syndrome, systemic sclerosis, and systemic lupus erythematosus [6]. OM is frequently associated with MAA such as anti-PM/Scl (anti-polymyositis/scleroderma), anti-SSA (anti–Sjögren’s-syndrome-related antigen A), and anti-U1-RNP (anti-U1 small nuclear ribonucleoprotein), found in other systemic autoimmune diseases [22]. ASS is characterized by myositis, Raynaud’s phenomenon, arthritis, mechanic’s hands, and interstitial lung disease (ILD). Presence of anti-Jo-1, the most prevalent antisynthetase antibody, is mostly associated with the classic triad of myositis, ILD, and arthritis, and is linked to a more favorable prognosis and lower mortality compared to other antisynthetase antibodies [23].

Polymyositis is currently considered the rarest IIM subtype [24,25] and some authors even question its existence [26]. It manifests with proximal weakness, elevated CK concentrations, myopathic electromyography (EMG) pattern, and inflammatory CD8 T cell infiltrates in histopathologic examination [6,11]. It is not associated with cutaneous changes, a family history of neuromuscular disease, or exposure to myotoxic drugs [25]. Many patients previously classified as having PM, have been reclassified to other forms of IIM such as ARS, IMNM, and IBM [6,27,28]. PM remains currently a diagnosis of exclusion [25,29] [Table 1].

**Table 1:** Characteristic clinical features, histopathological characteristics, and myositis-specific antibodies across different IIM subtypes. IIM = idiopathic inflammatory myopathy, DM = dermatomyositis, IBM = inclusion body myositis, IMNM = immune-mediated necrotizing myopathy, ASyS = antisynthetase syndrome, PM = polymyositis, MSA = myositis-specific antibodies, anti-Mi-2 = anti-nuclear helicase, anti-MDA-5 = anti-melanoma differentiation-associated gene 5, anti-NXP-2 = anti-nuclear matrix protein 2, anti-TIF-1γ = anti-transcriptional intermediary factor 1, anti-SAE = anti-small ubiquitin-like modifier-1 activating enzyme, anti-cN1A = anti-cytosolic 5′-nucleotidase 1A, anti-SRP = anti-signal recognition particle, anti-HMGCR = anti-3-hydroxy-3-methylglutaryl-coenzyme A reductase, anti-Jo-1 = anti-histidyl tRNA synthetase, anti-PL-7 = anti-threonyl-tRNA synthetase, anti-PL-12 = anti-alanyl-tRNA synthetase, anti-EJ = anti-glycyl-tRNA synthetase, anti-OJ = anti-isoleucyl-tRNA synthetase, anti-KS = anti-asparaginyl tRNA synthetase, anti-ZO = anti-phenylalanyl-tRNA synthetase, anti-Ha = anti-tyrosyl-tRNA synthetase.

IIM	Characteristic Clinical Features	Histopathological Characteristics	MSA
DM	Heliotrope rash, Gottron’s papules, Gottron’s sign, shawl sign, holster sign, mechanic’s hands, periungual redness and telangiectasias	Perimysial and perivascular infiltration of mainly B cells, CD4+ T cells, macrophages and dendritic cells, perifascicular atrophy, capillary depletion [30]	Anti-Mi-2, anti-MDA-5, anti-NXP-2, anti-TIF-1γ, and anti-SAE-1/2
IBM	Asymmetric pattern of weakness, involvement of finger flexors, wrist flexors, ankle dorsiflexors and knee extensors	CD 8+ T cell infiltrate of non-necrotic fibers, rimmed vacuoles, cytoplasmic protein aggregates, mitochondrial abnormalities	Anti-cN1A [31]
IMNM	Prominent muscle atrophy, severe proximal weakness, significantly elevated creatine kinase	Necrosis and regeneration of muscle fibers, scattered isolated CD68-prevalent cells, without CD8 invading or surrounding non-necrotic fibers [32]	Anti-SRP, anti-HMGCR
ASyS	Myositis, Raynaud’s phenomenon, arthritis, mechanic’s hands, interstitial lung disease	Perimysial fibrosis with endothelial lesion, perifascicular ischemia, necrotic fibers [33]	Anti-Jo-1, Anti-PL-7, Anti-PL-12, Anti-EJ, Anti-OJ, Anti-KS, Anti-Zo, Anti-Ha
PM	Proximal, symmetric weakness, diagnosis of exclusion	CD 8+ T cell infiltrate	

The estimated incidence for IIM ranges from 0.2 to 2 per 100,000 person-years, with prevalence from 2 to 25 per 100,000 people [34]. Although IIM are rare diseases, the economic, social, and mental health burden of the disease are substantial, surpassing other systemic autoimmune rheumatic diseases such as rheumatoid arthritis, systemic sclerosis, and systemic lupus erythematosus [35]. The cost of IIM is estimated at $52,210 per patient-year on average with nearly 1/3 of total costs being the result of loss of productivity of working-age individuals [35].

### 1.2. Current Treatment Options

According to 2022 British Society for Rheumatology guideline on management of pediatric, adolescent, and adult patients with idiopathic inflammatory myopathy, current IIM treatment should involve high dose corticosteroids as induction therapy, and disease modifying anti-rheumatic drugs (DMARDS) such as azathioprine, methotrexate, or cyclosporine to achieve clinical remission and to reduce steroid burden and muscle inflammation [Table 2] [36]. Non-pharmacological management consists of supervised exercise programs, assessment of psychological well-being, quality of life, and psychiatric comorbidities and addressing the adverse effects of steroids. Cancer risk should be assessed in all patients and screening is particularly advised in patients at older age at onset, of male gender with dysphagia, cutaneous necrosis, or resistance to immunosuppressive therapy, rapid disease onset, positive anti-TIF1-γ autoantibodies, positive anti-NXP2 autoantibodies, and negative for known myositis-specific autoantibodies [36].

Intravenous immunoglobulin, cyclophosphamide, rituximab, and abatacept should be considered for the treatment of refractory disease [36] [Table 2].

**Figure 1:**
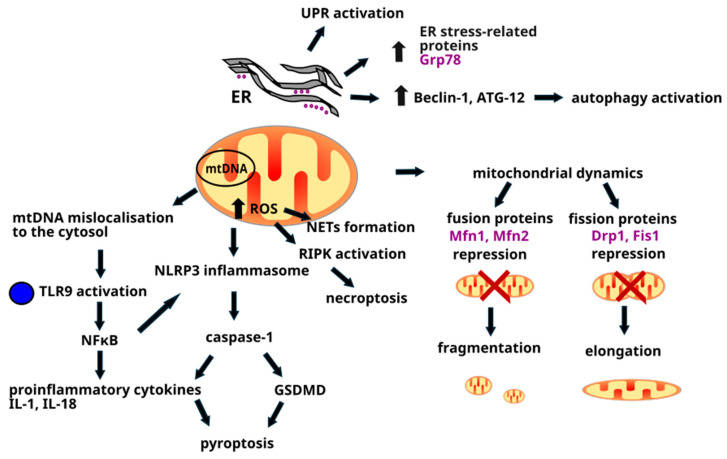
Molecular mechanisms underlying the pathogenesis of idiopathic inflammatory myopathy. ATG-12 = autophagy-related 12, Drp1 = dynamin-related protein 1, ER = endoplasmic reticulum, Fis1 = fission protein 1, GSDMD = gasdermin D, Grp78 = glucose-regulated protein 78, IL = interleukin, mtDNA = mitochondrial DNA, Mfn = mitofusin, NETs = neutrophil extracellular traps, NF-κB = nuclear factor kappa-light-chain-enhancer of activated B cells, NLRP = nucleotide-binding oligomerization domain, leucine rich repeat and pyrin domain containing, RIPK = receptor-interacting protein kinase, ROS = reactive oxygen species, TLR9 = toll-like receptor 9, UPR = unfolded protein response.

Although the main mechanisms underlying inflammatory myopathies is thought to be the autoimmune aggression against muscle cells, the lack of response after immunosuppression treatment in some patients and poor correlation between muscle weakness and inflammatory cells infiltration in muscle biopsies [37,38] suggest that other nonimmune mechanisms are involved in the pathogenesis of IIM [Figure 1]. Exploring specific molecular pathways underlying IIM development may help identify novel therapeutic targets improving IIM treatment efficacy and paving the way to personalized therapy.

This paper will discuss the role of mitochondria, ER stress, myokines, cell death, and neutrophil dysregulation in IIM pathophysiology, as well the newest therapeutic strategies targeting these mechanisms. As physical therapy has been demonstrated to be safe and effective in this group of patients [39,40,41,42,43,44], constituting an essential part of IIM management [Table 2], additional focus will be directed towards molecular mechanisms underlying the effects of exercises in myositis treatment.

## 2. Discussion

### 2.1. Mitochondrial Dysfunctions

Although mitochondria are known mainly for generating energy in a cell, their function in human organisms is much more complex as they play a key role in the inflammatory and autoimmune response. Mitochondrial components such as mitochondrial DNA (mtDNA) can act as damage-associated molecular patterns (DAMPs) activating the inflammatory signaling via the cyclic GMP-AMP synthase-cyclic GMP-AMP-stimulator of interferon genes (cGAS-cGAMP-STING) pathway [45,46,47]. Leaked mtDNA can also activate the nucleotide-binding oligomerization domain, leucine rich repeat, and pyrin domain containing (NLRP) inflammasome that stimulates caspase-1, which proteolytically cleaves pro-inflammatory cytokines: IL-1β and IL-18, as well as pore-forming gasdermin D (GSDMD) [46,47] [Figure 1]. Furthermore, they participate in T cell activation and differentiation [46,48,49]. Activation of inflammatory pathways by mitochondria is emerging as a key area of focus in myositis and opens new avenues for targeted therapy in chronic muscle inflammation.

Targeting mitochondrial dynamics may be a valuable approach in treating muscle inflammatory diseases. It was demonstrated that mitochondrial dynamics control the activation of intracellular inflammatory pathways, as repression of the mitochondrial fusion proteins mitofusin-1 (Mfn1) and mitofusin-2 (Mfn2) induces mitochondrial fragmentation and Toll-like receptor 9 (TLR9)-dependent nuclear factor kappa-light-chain-enhancer of activated B cells (NFκB) activation. NFκB is a transcription factor that induces the expression of pro-inflammatory genes encoding cytokines and chemokines and regulates the survival, activation, and differentiation of innate immune cells and inflammatory T cells [50]. Moreover, repression of fission proteins dynamin-related protein 1 (Drp1) and fission protein 1 (Fis1) caused mitochondrial elongation, and both NFκB-dependent and type I interferon (IFN) inflammatory responses, accompanied by mislocalization of mtDNA to the cytosol. mtDNA mislocalization and its cytopasmatic accumulation can trigger inflammation via the cyclic cGAS-STING pathway, observed in many neurodegenerative diseases [51]. This indicates that mitochondrial stress can trigger sterile inflammatory responses and targeting mitochondrial dynamics could be a valuable therapeutic strategy in IIM [52] [Figure 1].

Basualto-Alarcón demonstrated that mitochondria isolated from human derived IIM myoblasts were functional and exhibited plasticity as they could respond to metabolic stress by increasing their respiration. However, IIM-derived cells generated increased amounts of reactive oxygen species (ROS) compared to control cells, and under an additional oxidative stimulus (hydrogen peroxide), ROS-mediated cell death significantly increased. This indicates that IIM-derived cells were more prone to oxidative damage and that the processes required for adaptation may ultimately be detrimental to cell survival. Given the preserved mitochondrial plasticity yet increased susceptibility to oxidative damage in IIM, unraveling the balance between ROS production and ROS scavenging may have important therapeutic implications [53].

Increased level of IFN represents another possible mechanism of mitochondrial impairment in myositis. Interferons are cytokines playing a key role in antiviral response and immune modulation [54]. They are classified into three main types: type I (IFN-α and IFN-β), type II (IFN-γ), and type III (IFN-λ). Upregulation of IFN-induced genes and hyperactivation of IFN signaling has been found in various subtypes of IIM [55,56], and IFN-targeted antibodies that reduce IFN activity have shown a promising treatment in IIM [57,58]. Predominant type I IFN response has been shown in DM, whereas type II IFN are more characteristic for IBM and ASyS [54]. IFN-β was demonstrated to increase reactive oxygen species production and induce mitochondrial damage in human myotubes from patients with DM [59]. Mitochondrial dysfunctions were in turn shown to increase ROS production that trigger the expression of type I IFN-inducible gene and muscle inflammation, thus self-sustaining the disease. Importantly, ROS scavenging was demonstrated to prevent mitochondrial dysfunctions, type I IFN signature, inflammatory cell infiltration, as well as muscle weakness in an experimental autoimmune myositis murine model, suggesting that targeting mitochondria abnormalities may be a promising therapeutic strategy [59]. In line with this conclusion is the study on the role of type II interferon in myositis. Exposure of differentiating human myoblasts to IFNγ reduced the expression of mitochondrial genes responsible for oxidative phosphorylation and altered mitochondrial ultrastructure in murine model. Similar to Meyer’s experiment, ROS scavenging reduced muscle cell infiltration and improved locomotor activity, grip strength, muscle strength as well as resulted in fewer abnormal mitochondria than in untreated mice, implying once again that there is a dynamic interplay between mitochondrial dysfunction and the development of inflammation in IM [60].

So far, therapies targeting oxidative damage have shown some favorable outcomes in in vitro studies of human myoblasts [60] and experimental autoimmune myositis murine models [59]. Treatment with the antioxidant *N*-acetylcysteine reduced cellular and oxidative stress and improved overall motor phenotype and functions. Butylphthalide was found to increase the superoxide dismutase and catalase activity, enhance ATPase activity in muscle mitochondrial membranes and reduce the number of apoptotic cells in a guinea pig model [61,62].

Anti-mitochondrial antibodies (AMAs), although rarely found in patients with IIM [63], have recently gained attention for its association with severe cardiac involvement including cardiac arrest, refractory ventricular tachycardia and heart failure [64,65,66,67,68]. AMAs are classified among the MAA. They target the E2 subunits of the 2-oxo acid dehydrogenase complexes inside the inner mitochondrial membrane and are principal biomarkers for primary biliary cholangitis (PBC), found in 90–95% of patients. Increased cardiac involvement may be partially attributed to the presence of PBC as it is associated with elevated cardiovascular risk [69]. It is suggested that AMA-positive IIM constitutes a distinct IIM phenotype with mild striated muscle involvement and severe cardiac manifestations. [63,64]. AMAs have a low sensitivity and high specificity for the diagnosis of cardiac involvement of IIM patients [70]. Their presence may serve as a warning sign for potential cardiovascular damage [70], thus screening for cardiac diseases in patients with IIM is recommended in case of the detection of AMA [64,71].

One of the latest therapeutic interventions in inflammatory myopathies involves mitochondrial transplantations. In a comprehensive clinical trial, Kim et al. demonstrated that transplantations of mitochondria isolated from umbilical cord mesenchymal stem cells into myoblasts derived from IIM patients enhanced muscle differentiation and mitochondrial function [72]. Moreover, an anti-inflammatory effect was demonstrated in myositis murine model, shown by reduced interleukin 6 (IL-6) and tumor necrosis factor α (TNF-α) mRNA expression. Furthermore, muscle regeneration capacity was improved as the decreased myofiber cross-sectional area and expression of mitochondrial proteins—translocase of outer mitochondrial membrane 20 (TOM20) and succinate dehydrogenase subunit B (SDHB) in murine quadriceps—was restored after mitochondrial treatment. Interestingly, a positive effect on metabolic distortions in muscle tissue has also been demonstrated as mitochondrial transplantation restored the reduced malate/aspartate ratio in a murine model. In in vitro model, such treatment prevented cell death induced by perforin and granzyme B by inhibiting the increase in pro-apoptotic cytochrome C, caspase-3, and caspase-9; moreover, it also attenuated the decreased expression of myogenic differentiation 1 (MyoD) and myogenin, crucial for myoblast proliferation. Most importantly, such transplantations demonstrated no severe adverse drug reactions and showed improvement on manual muscle testing (MMT) in patients with PM and DM.

The effect of physical exercise on mitochondrial dysfunction has been shown in a rat model of sporadic inclusion body myositis [73]. It was demonstrated that resistance training enhanced mitochondrial function by improving oxidative capacity and mitochondrial quality control (MQC)—a group of processes involving biogenesis, mitochondrial dynamics, and mitophagy, crucial for preserving their integrity and function [74,75,76] via reducing amyloid-β accumulation. Aβ is a protein that plays a key role in the pathogenesis of neurodegenerative diseases such as, primarily, Alzheimer’s disease, where it aggregates and forms amyloid plaques in the brain disrupting neuronal function [77]. It has also been found to deposit within muscle fibers in IBM, constituting a pathological hallmark in this IIM subtype [78]. Importantly, Aβ can accumulate in mitochondrial matrix and disrupt the transport of proteins necessary for mitochondrial function [63,79,80]. Muscle cells derived from IIM patients after 6 months of intensive training were presented with normalized distribution patterns of OxPHOS proteins, increased fat oxidative capacity and reduced proportion of non-oxidative fatty acid metabolism indicating enhanced mitochondrial efficiency [81].

### 2.2. Endoplasmic Reticulum Stress

Endoplasmic reticulum (ER) stress refers to the accumulation of unfolded and misfolded proteins in the ER lumen. The protein folding capacity can be disrupted by oxidative stress, hypoxia, Ca^2+^ alterations, and inflammatory stressors [82,83]. The activated unfolded protein response (UPR) is mediated by three transmembrane proteins: protein kinase R (PKR)-like endoplasmic reticulum kinase (PERK), inositol-requiring protein 1α (IRE1α), and activating transcription factor 6 (ATF6). Glucose-regulated protein 78/immunoglobulin-binding protein (GRP78/BiP) is a master regulator of the unfolded protein response [84].

Vidal et al. points to the crucial role of RyR1 depletion-induced ER stress in the pathogenesis of myopathies. Decreased ryanodine receptor type 1 (RyR1) protein levels lie at the core of recessive *RYR1*-related myopathies, a class of congenital myopathies. However, a significant decrease in RyR1 protein levels was also found in muscle samples of patients with inflammatory myopathies. Interestingly, the RyR1 protein depletion impaired Ca^2+^ transfer from the endoplasmic reticulum to the mitochondria by reducing the ER–mitochondria contact length and resulted in the accumulation of elongated mitochondria that evade degradation [85,86,87]. Moreover, there was an increase in ER stress markers such as GRP78/Bip and DNA damage-inducible transcript 3 (DDIT3) in IM muscle samples which suggests that RyR1 protein depletion may cause endoplasmic reticulum stress in IIM [87].

Ma et al. suggested that ER stress-mediated activation of autophagy is involved in pathogenesis of idiopathic inflammatory myopathies [88]. It was demonstrated that the expression of GRP78/BiP, an ER chaperone protein and autophagy-related 12 (ATG12) protein and Beclin-1, involved in autophagy pathways, was elevated in skeletal muscles of patients with dermatomyositis and immune-mediated necrotizing myopathy. Moreover, the expression of proteins involved in UPR pathway and autophagy was increased in IMNM. Importantly, ER stress correlated with muscle weakness in immune-mediated necrotizing myopathy subtype. Paradoxically, although the study showed a detrimental role in ER stress pathway in IIM, it has also been linked with beneficial processes such as myofiber regeneration in IMNM and DM. This suggests that ER stress could have multiple roles in regulating disease progression of IIM.

Interestingly, the increased expression of ER stress-related proteins such as Grp78, Grp94, and PERK in autoimmune myopathy murine model has been attenuated by high-intensity interval training (HIIT). Furthermore, HIIT was also demonstrated to increase the expression of mitochondrial respiratory complexes, restoring muscle function [89]. Overall, HIIT has been shown to influence mitochondria oxidative capacity and the activation of ER stress-dependent pathway improving fatigue resistance in a murine model.

Similarly, high-force eccentric contractions training prevented the reduction in force and suppressed the upregulation of ER stress proteins such as Grp78 and Grp94 in a murine model. Moreover, it also enhanced the expression and myofibrillar binding of small heat-shock proteins (sHSPs) which stabilize myofibrillar structure and function, suggesting that resistance training involving high-force eccentric contractions can effectively protect against the muscle weakness in IIM mice by reducing ER stress- and sHSP-dependent pathways [90].

### 2.3. Cell Death: Necroptosis, Pyroptosis, Apoptosis, and FAP Senescence

Necroptosis is a form of regulated cell death induced by the activation of death receptors and mediated by the necrosome which involves receptor-interacting protein kinase 1 and 3 (RIPK1, RIPK3) and mixed-lineage kinase domain-like protein (MLKL) [91]. It stimulates the inflammatory response by releasing damage-associated molecular pattern molecules (DAMPs) such as high-mobility group box 1 (HMGB1) [92,93,94].

Peng et al. found that the expression of RIPK3 and MLKL and their phosphorylated forms was substantially increased in muscle tissue of IIM patients. Moreover, a significant correlation of RIPK3 and MLKL expression with the severity of clinical and pathologic muscle damage was demonstrated. Importantly, inhibition of necroptosis with necrostatin-1 and knockdown of MLKL expression successfully prevented cells from undergoing necroptosis-induced cell death, suggesting that targeting necroptotic pathways could be a noteworthy therapeutic strategy in inflammatory myopathy treatment [95].

Kamiya et al. demonstrated that targeting FAS ligand-dependent necroptosis in muscle fibers ameliorates inflammatory myopathies. Dying muscle fibers release high levels of HMGB1 leading to the exacerbation of muscle inflammation and subsequent muscle injury in PM [96]. Necroptosis could be targeted via glucagon-like peptide-1 receptor (GLP-1R) agonists, anti-diabetic drugs with anti-inflammatory, and cell death protective properties. GLP-1 receptor is expressed on the plasma membrane of the inflamed muscle fibers. Stimulation of GLP-1R with a GLP-1R agonist suppresses myotube necroptosis and the release of inflammatory cytokine mediators such as HMGB1. The key mechanisms involved in this process include suppressing the accumulation of ROS and downregulating phosphoglycerate mutase family member 5 (PGAM5), a mitochondrial protein involved in necroptosis of the myotubes which led to a rapid improvement in muscle weakness in a PM murine model [97].

Moreover, Mo et al. demonstrated that the caspase-4/5/11-mediated non-canonical pyroptotic pathway may be involved in the pathogenesis of inflammatory myopathy. Higher expression levels of the caspase-4, caspase-5, and caspase-11 proteins were detected in the murine model of experimental autoimmune myositis compared to the control groups. Drugs such as glyburide, a sulfonylurea used in diabetes treatment, and brilliant blue G, an antagonist of P2X purinergic receptor 7 (P2X7) receptors, have been shown to inhibit the activation of NLRP3 and reduce the secretion of pro-inflammatory cytokines [98].

An interesting insight into the effects of exercises on cell death pathways in inflammatory myopathy has been reported by Saito et al. [99]. It was demonstrated that senescence of fibro-adipogenic progenitors (FAPs)—regulators of muscle stem cell function [100]—induces muscle regeneration in response to exercise. However, surprisingly, in chronic inflammatory myopathy murine model, an impaired FAP senescence was observed after exercises. Failure of FAPs to enter a senescent state led to muscle degeneration and FAP accumulation due to cells’ shift towards an anti-apoptotic and pro-fibrotic phenotype. Targeting pro-senescent interventions with exercise and AMP-activated protein kinase stimulation reversed FAP resistance against tumor necrosis factor-mediated apoptosis. However, a different study indicated that senescent FAPs in muscle negatively impacted the function of muscle stem cells and that eliminating senescent FAPs improved muscle strength and reduced fibrosis [101]. Recent advances in single-cell RNA sequencing revealed the cellular heterogeneity of FAPs and their intricate regulatory network in different stages of muscle regeneration. Targeting FAPs to limit their excessive accumulation and deregulation of cell activity is a new therapeutic strategy that may reduce fibrofatty deposition and promote muscle regeneration in muscular disorders [100].

### 2.4. Myokines

Recent findings suggest that muscles play not only a locomotor role in human organisms but act also as a secretory, immunological organ, releasing cytokines, chemokines, and small peptides, known as myokines [37], in response to inflammatory stimuli or physical exercise [102]. This secretome exerts autocrine function modulating muscle metabolic processes, but it can also act on surrounding and distant organs such as bones, adipose tissue, liver, pancreas, and brain, exerting paracrine and endocrine effects [37,103,104].

Activin A and myostatin are myokines belonging to the TGF-*β* (transforming growth factor-beta) family which are potent inhibitors of muscle growth. Follistatin is their strong antagonist and can increase muscle mass and strength. However, interestingly, clinical trial on the use of bimagrumab, a monoclonal antibody against activin type II receptors that prevents binding of activin and myostatin, did not provide clinical benefits in terms of improvement in mobility in patients with inclusion body myositis [105]. Vernerova et al. suggest that the alterations in the whole activin A-myostatin-follistatin system rather than a single myokine underlie the IIM pathophysiology. Lower myostatin and higher follistatin levels were reported in IIM in comparison to healthy subjects, suggesting that upregulated muscle growth-related and downregulated atrophy-related molecules may be a compensatory mechanism in the disease [38].

The major histocompatibility complex (MHC) I protein which presents endogenous peptide fragments on the cell surface, subsequently recognized by cytotoxic T cells, is overexpressed in skeletal muscles in IIM [106,107,108]. Thoma et al. found that MHC I overexpression can induce pro-inflammatory cytokine and chemokine release from myoblasts in in vitro conditions. Media from MHC I overexpressing cells induced T cell chemotaxis, mitochondrial dysfunction through increased proton leak and atrophy of myotubes. Interestingly, these effects were alleviated in the presence of salubrinal, an agent playing an important role in translational attenuation which suggests that myokines release is mediated by the nonimmune endoplasmic reticulum stress pathway [108]. Moreover, Batthari et al. demonstrated that immunoproteasomes, protein complexes responsible for MHC I antigen presentation and protein degradation of cells exposed to oxidative stress [109], are key to regulating myokines in IIM [110]. Inhibition of a β5i immunoproteasome subunit amplified TNF-α and IFN-γ-mediated myokine expression, suggesting that proteasome is crucial in maintaining myokines homeostasis.

### 2.5. Neutrophil Dysregulation

Neutrophil extracellular traps (NETs) are fibrous structures composed of chromatin, histones, and myeloperoxidase, that play a crucial role in infections, binding, and eliminating microbes by exposing them to high concentrations of nuclear and granular proteins [111,112,113,114]. However, since their discovery in 2004 [115], they have also been implicated in other, non-infectious processes, such as prolonged inflammatory response and tissue damage, thus exhibiting a dual role in various pathologies. In several studies, NETs have been proposed to be a new type of DAMPs [116,117,118]. Similar to damage-associated molecular patterns, they are released in response to injury or infection, subsequently activating the innate immune response. NETs components including HMGB-1, DNA, histones, extracellular matrix, and serum amyloid A, by acting on different immune cells, promote the release of cytokines, activate the inflammasome and, consequently, exacerbate the inflammatory response [117]. Prolonged production and improper degradation of NETs have been found in a variety of autoimmunological disorders including systemic lupus erythematosus, rheumatoid arthritis, anti-neutrophil cytoplasmic antibodies-associated vasculitis, and inflammatory myopathy [119,120].

By examining DM/PM patients’ plasma, Zhang et al. found that they exhibited a significantly enhanced capacity for inducing NETs, measured by elevated levels of circulating cell-free DNA and LL-37, an antimicrobial peptide and an important component of NET [121]. Moreover, it was shown that, in comparison to healthy subjects, NETs could not be completely degraded due to the reduction in DNase I activity, especially in DM/PM patients with interstitial lung disease [122]. Seto et al. demonstrated that NETs were associated with clinical manifestations in IIM patients and specific MSAs. Anti-MDA5, a myositis-specific antibody associated with dermatomyositis, was shown to directly enhance NET formation. NETs were found in affected muscles, skin, and lungs in DM subjects who were positive for MSAs but not in MSA-negative IIM, which may present valuable therapeutic implementations. Importantly, NETs were demonstrated to directly disrupt muscle tissue, as exposure to already formed myotubes showed increased cell death through a mechanism mediated by citrullinated histones present in NETs [123].

As DNase activity I is decreased in patients with IIM- ILD leading to overproduction of NETs, pulmonary vascular endothelial cells damage, and lung fibroblast proliferation [124,125], novel therapeutic strategies have been extensively researched in the context of IIM-ILD. Feng et al. found that NETs promote alveolar epithelial–mesenchymal transition, accelerating the development of ILD and that the cyclic cGAS-STING signaling pathway is an underlying mechanism of action. In vitro and in vivo studies showed that STING inhibitors prevented the epithelial–mesenchymal transition and reduced the inflammatory response in the murine model, suggesting that targeting the cGAS–STING pathway may be a valuable therapeutic option [118]. Zhao et al. found that NETs induce pyroptosis of pulmonary microvascular endothelial cells by activating the NLRP3 inflammasome [116] and Li et al. demonstrated that colchicine inhibited inflammasome activation and endothelial cell pyroptosis, inhibiting NETs formation in experimental autoimmune myositis model mice [126].

### 2.6. Interplay Between Immune and Nonimmune Mechanisms

The immune and nonimmune processes do not act independently but are rather a part of coordinated network. Reactive oxygen species participate in immune cell infiltrate which increases ROS formation and exacerbates ROS-mediated damage in muscles [127,128]. Moreover, they induce NETs formation by causing oxidative DNA damage which activates DNA repair mechanisms contributing to chromatin decondensation and facilitating NET release [129,130,131,132,133]. Furthermore, increased ROS levels have been implicated in the activation of pyroptotic and necroptotic pathways by activating NLRP3 inflammasome, NF-κB pathway, and RIPK1 and RIPK3 activation [134,135,136,137,138] [Figure 1]. Meyer et al. suggest that these are ROS that are at the crossroad between immune and nonimmune cell mediated mechanisms and that targeting their excessive production should be a primary focus of IIM therapeutic interventions [138].

However, an interesting relationship was observed also between endoplasmic reticulum and immune mechanisms. Overexpression of MHC I can induce ER stress which in turn has been observed to sustain MHC I overexpression and upregulate muscle-derived pro-inflammatory cytokines [106,108,139,140]. Endoplasmic reticulum acts closely with proteasome in protein degradation and MHC I antigen presentation. Immunoproteasome can induce MHC I overexpression and is responsible for myokine production and myokines-mediated attraction of immune cells in muscle fibers [110].

Interestingly, Lightfoot et al. stated that inflammatory cell infiltrations should be regarded rather as a secondary phenomenon in IIM as muscle dysfunction continues even after suppression of inflammation and atypical myokine production resulting from MHC-I–induced ER stress appears to be the primary pathological event associated with chemoattraction of immune cells, ultimately contributing to disease progression [37]. Other authors indicate that both immune and nonimmune mechanisms are critical in IIM pathogenesis and their involvement vary in different IIM types [141,142,143,144,145]. Evidence for nonimmune mediated mechanisms is most robust in the case of inclusion body myositis [146], where immunosuppressive treatment has consistently shown limited or no clinical benefit [147,148] and mitochondrial abnormalities are part of diagnostic criteria [149] with growing recognition of their significance [150]. Nonetheless, ER stress, mitochondrial dysfunctions as well as the presence of AMA are being increasingly recognized also in other IIM types, particularly dermatomyositis [151]. Gonzalez-Chapa predicts that AMA-positive IIM may soon be set apart as a distinctive type of DM [63]. Notably, growing in understanding of different molecular pathways underlying IIM pathophysiology and the identification of new autoantibodies may refine the current classification of inflammatory myopathies, as it has already happened in the case of polymyositis [27,152].

## 3. Challenges and Future Prospects

Large heterogeneity of this group of disorders, misdiagnosis stemming from overlapping symptoms with other rheumatic diseases, and low prevalence limiting the number of subjects in clinical trials constitute the main challenges in IIM research. Nonetheless, an increasing number of clinical trials [72,105,153,154,155,156,157,158] holds promise for finding a link between molecular changes in myositis and the development of effective therapeutic strategies.

Another limitation is that many studies involving animal models or in vitro experiments often performed on murine cell lines, which present several translational challenges. Furthermore, the overexpression of immune proteins such as MHC I was induced artificially [108]. In human myositis, a constellation of interacting biological processes appears to drive disease manifestation rather than a single factor. Intricate cellular interactions in inflammatory pathways may not be properly mirrored in murine cell lines. More studies on human cells as well as more randomized clinical trials are needed to further elucidate IIM pathogenesis. Importantly, novel diagnostic tools including transcriptomics, proteomics, and machine learning may help uncover molecular signatures contributing to inter-patient variability supporting the advancement of precision medicine approaches [159,160,161,162,163].

**Figure 2:**
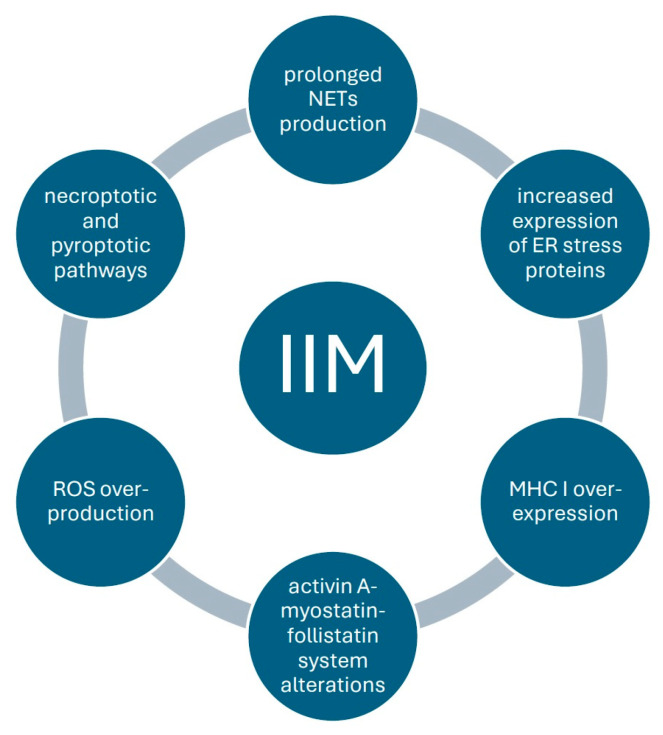
Emerging therapeutic targets in the treatment of idiopathic inflammatory myopathy.

Although an effective IIM treatment has not yet been found, recent innovative therapies targeting NETs formation, ROS overproduction, and pyroptotic and necroptotic pathways together with mitochondrial transplantations [Figure 2] hold promise for improving muscle function and alleviating inflammation in this group of patients. Molecular mechanisms underlying the effects of exercises such as decreased ER stress, modulation of protein synthesis, and improved oxidative capacity indicate that physical therapy, the simplest, safest, and most accessible form of treatment, should constitute an integral part of IIM management.

## Figures and Tables

**Table 2:** Idiopathic inflammatory myopathy management. DMARDs = disease modifying anti-rheumatic drugs, IVIG = intravenous immunoglobulin.

Idiopathic Inflammatory Myopathy Management		
Pharmacological	Non-Pharmacological	Refractory Disease
Glucocorticoids	Exercise program	IVIG
DMARDs (azathioprine, methotrexate, cyclosporine, tacrolimus)	Psychological well-being and quality of life assessment	Cyclophosphamide
	Addressing steroid adverse effects	Rituximab
	Cancer screening	Abatacept

## Data Availability

Not applicable.

## Abbreviations

The following abbreviations are used in this manuscript:

AMA	Antimitochondrial antibodies
ASyS	Antisynthetase syndrome
ATF6	Activating transcription factor 6
ATG12	Autophagy related 12
cGAS	Cyclic GMP-AMP synthase
cGAMP	Cyclic GMP-AMP
CK	Creatine kinase
cN1A	Cytosolic 5′-nucleotidase 1A
DDIT3	DNA damage-inducible transcript 3
DM	Dermatomyositis
DMARDs	Disease modifying anti-rheumatic drugs
Drp1	Dynamin-related protein 1
eIF2α	Eukaryotic translation initiation factor 2 subunit alpha
EMG	Electromyography
ER	Endoplasmic reticulum
Fis1	Fission protein 1
GLP-1	Glucagon-like peptide-1
GRP78/BiP	Glucose-regulated protein 78/binding immunoglobulin protein
GSDMD	Gasdermin D
HIIT	High-intensity interval training
HMGβ1	High-mobility group box 1
HMGCR	3-hydroxy-3-methylglutaryl-coenzyme A reductase
IBM	Inclusion body myositis
IFN	Interferon
IIM	Idiopathic inflammatory myopathy
ILD	Interstitial lung disease
IMNM	Immune-mediated necrotizing myopathy
IVIG	Intravenous immunoglobulin
IL	Interleukin
MAA	Myositis-associated autoantibodies
MDA-5	Melanoma differentiation-associated gene 5
Mfn	Mitofusin
MHC-1	Major histocompatibility complex class I
MLKL	Mixed lineage kinase domain-like protein
MSA	Myositis-specific autoantibodies
mtDNA	Mitochondrial DNA
MyoD	Myogenic differentiation 1
NETs	Neutrophil extracellular traps
NF-κB	Nuclear factor kappa-light-chain-enhancer of activated B cells
NLRP	Nucleotide-binding oligomerization domain, Leucine rich Repeat and Pyrin domain containing
NXP-2	Nuclear matrix protein 2
OM	Overlap myositis
OxPHOS	Oxidative phosphorylation
P2X7	P2X purinergic receptor 7
PBC	Primary biliary cholangitis
PERK	Protein kinase RNA-like endoplasmic reticulum kinase
PGAM5	Phosphoglycerate mutase family member 5
PM	Polymyositis
RIPK	Receptor-interacting protein kinase
ROS	Reactve oxygen species
RyR1	Ryanodine receptor 1
SAE	Small ubiquitin-like modifier-1 activating enzyme
SDHB	Succinate dehydrogenase subunit B
sHSP	Small heat shock proteins
SRP	Signal recognition particle
STING	Stimulator of interferon genes
TGFβ	Transforming growth factor beta
TIF-1γ	Transcriptional intermediary factor 1
TLR9	Toll-like receptor 9
TNFα	Tumor necrosis factor-alpha
TOM20	Translocase of outer mitochondrial membrane 20
UPR	Unfolded protein response
